# Supplementary material for: The concentration and energetic content of floral nectar sugars: calculation, conversions, and common confusions
Source: J Pollinat Ecol. Author manuscript; Available in PMC 2026 Mar 23. (PMC7618919; doi:10.26786/1920-7603(2025)821)
Supplement: Supplementary Information [file EMS212872-supplement-Supplementary_Information.pdf]

Supplementary Information for: The concentration and energetic content of floral nectar sugars: calculation, conversions, and common confusions

Jonathan G Pattrick[1,*], Jennifer Scott[1], Geraldine A Wright[1]

[1]Department of Biology, University of Oxford, John Krebs Field Station, Wytham, Oxford, OX2 8QJ

1. Calculating the energetic content of sucrose, glucose, and fructose.

The most common values given for the energetic content of nectar sugars are those derived from the enthalpy of combustion ($\Delta_c H°$) for each of these compounds (Brown et al. 2008; Schmid et al. 2011; Balfour et al. 2015). The enthalpy of combustion can be calculated from the enthalpy of formation using Hess's law and the known enthalpy of formation ($\Delta_f H°$) for the combustion products (CO_2 and H_2O). These are directly available from the CRC Handbook of Chemistry and Physics (Lide 2004) with $\Delta_f H°$ for sucrose = -2226.1, glucose = -1273.3, fructose = -1265.6, CO_2 = -393.510 and H_2O = -285.830 kJ/mol, all at a temperature of 298.15 K (Lide 2004).

Hess's law states that $\Delta_c H° = \sum(\Delta_f H°$ of the products$) - \sum(\Delta_f H°$ of the reactants$)$ (Lide 2004).

For fructose and glucose the combustion reaction is:

$C_6H_{12}O_6 + 6O_2 \rightarrow 6CO_2 + 6H_2O$

And for sucrose the combustion reaction is:

$C_{12}H_{22}O_{11} + 12O_2 \rightarrow 12CO_2 + 11H_2O$

Hence for the three sugars the enthalpy of combustion can be calculated as follows:

$\Delta_c H°$ (sucrose) = $[12(-393.51) + 11(-285.83)]-(-2226.1)$ = -5640.15 kJ / mol

$\Delta_c H°$ (glucose) = $[6(-393.51) + 6(-285.83)]-(-1273.3)$ = -2802.74 kJ / mol

$\Delta_c H°$ (β-D-fructose) = $[6(-393.51) + 6(-285.83)]-(-1265.6)$ = -2810.44 kJ / mol

Energy in kJ / mol can be converted to Calories (kcal) as there are 4.184 kJ per kcal (using the definition for the thermochemical calorie) (Lide 2004).

2. Converting between % w/w and % w/v (g / 100 ml solution) for solutions with multiple sugars

Here we give worked examples for converting between % w/w and % w/v (g / 100 ml solution) for sugar solutions containing multiple sugars, using equations 4 and 5 from the main text.

As an example for converting to % w/v from % w/w, consider a nectar which contains 10% w/w sucrose, 15% w/w glucose and 12% w/w fructose. The total sugar content (in g) in 100 g of solution is equivalent to $C_{w\ combined} = 10 + 15 + 12 = 37$ and, using Equation 4:

for sucrose, $C_{w\ sugar} = 10$, therefore $C_{v\ sugar} = 10 \times (0.998709 + 3.7430\mathrm{E}^{-3} \times 37 + 1.7639E^{-5} \times 37^2) = 11.6\%\ \mathrm{w/v}$,

for glucose, $C_{w\ sugar} = 15$, therefore $C_{v\ sugar} = 15 \times (0.998709 + 3.7430\mathrm{E}^{-3} \times 37 + 1.7639E^{-5} \times 37^2) = 17.4\%\ \mathrm{w/v}$.

For fructose, $C_{w\ sugar} = 12$, therefore $C_{v\ sugar} = 12 \times (0.998709 + 3.7430\mathrm{E}^{-3} \times 37 + 1.7639E^{-5} \times 37^2) = 13.9\%\ \mathrm{w/v}$.

Here, as % w/v is equivalent to g / 100 ml (and therefore mg / 100 µl) of solution, these values can then be used in combination with Table 4 (i.e. 16.48, 15.56 and 15.60 J mg^{-1} for sucrose, glucose and fructose respectively) to work out the energetic content per µl of this nectar.

$$\frac{16.48 \times 11.6 + 15.56 \times 17.4 + 15.60 \times 13.9}{100} = 6.80\ \mathrm{J}/\mu\mathrm{l}$$

As an example for converting to % w/w from % w/v, consider a nectar which contains 20% w/v

sucrose, 14% w/v glucose and 18% w/v fructose. The total sugar content (in g) in 100 ml of solution is equivalent to $C_{v\ combined} = 20 + 14 + 18 = 52$ and, using Equation 5:

for sucrose, $C_{v\ sugar} = 20$, therefore , $C_{w\ sugar} = \frac{20}{0.998138 + 3.8765E^{-3} \times 52 - 1.8716E^{-6} \times 52^2} = 16.7\%\ \mathrm{w/w}$,

for glucose, $C_{v\ sugar} = 14$, therefore , $C_{w\ sugar} = \frac{14}{0.998138 + 3.8765E^{-3} \times 52 - 1.8716E^{-6} \times 52^2} = 11.7\%\ \mathrm{w/w}$,

for fructose, $C_{v\ sugar} = 18$, therefore , $C_{w\ sugar} = \frac{18}{0.998138 + 3.8765E^{-3} \times 52 - 1.8716E^{-6} \times 52^2} = 15.1\%\ \mathrm{w/w}$.

As noted in the main text, the values calculated for each individual sugar in these conversions will vary depending on the concentration of the other sugars. For example, given a nectar which is 20% w/w sucrose, 20% w/w glucose, and 20% w/w fructose, the sucrose concentration of this nectar when converted to % w/v will be 25.74% w/v. However, for a similar nectar but with no fructose in (i.e. 20% w/w sucrose, 20% w/w glucose and 0% w/w fructose), the sucrose concentration will be 23.53% w/v.

3. Methods for reporting sugar concentration in % w/v

Concentration reported as % w/v can refer to grams of solute in 100 ml of *solution* or, grams of solute in 100 ml *solvent* (Bolten et al. 1979). For clarity, a 20% w/v (g solute / 100 ml solution) sucrose solution would be made by diluting 20 g of sucrose in water, made up to a final solution volume of 100 ml. Meanwhile, a 20% w/v (g solute / 100 ml solvent) sucrose solution would be made by adding 20 g of sucrose to 100 ml of water. For this second approach the final solution volume would therefore be >100 ml.

For very dilute solutions (e.g. < 0.1%) the difference between the two approaches is minimal. However, pollination ecology often concerns high solute concentrations, where the sugar forms a significant proportion of the total volume of the solution.

Although % w/v (g solute / 100 ml solution) would seem to be the more commonly used measure (Lide 2004); we assessed this with a small literature search. Using Google Scholar we used the search terms "%, w/v, nectar", and then "%, w/v, nectar, pollinator" selecting the first 50 peer-reviewed papers (25 from each search, but avoiding replications) published from 2014 – 2024. We then classified the papers based on the method used for reporting concentration. Sometimes the method wasn't explicitly stated, but could be inferred.

Out of the 50 papers, we classified 10 as using % w/v (g solute / 100 ml solution), whereas one used % w/v (g solute / 100 ml solvent). We were unable to distinguish which of the two methods was used for the remaining 39 papers. This suggests that % w/v (g solute / 100 ml solution) is indeed more common, but not the only approach used. We therefore we recommend explicitly stating the approach used.

For completeness, we also include equations to convert from % w/v (g sugar / 100 ml *solvent*) to the other measures considered. Concentration in % w/v (g sugar / 100 ml solvent) can be converted to % w/w using SI Equation 1:

Converting to % w/w from % w/v (g sugar / 100 ml solvent)

$$C_w = \frac{C_{vsolvent}}{100 + C_{vsolvent}} \times 100, \qquad \text{SI Equation 1}$$

where $C_{vsolvent}$ is the concentration in % w/v (g sugar / 100 ml solvent) and C_w is the concentration in % w/w, as in the main text. This can be converted to % w/v (g sugar / 100 ml *solution*) or other measures using the equations in the main text. The reverse conversion is:

Converting to % w/v (g sugar / 100 ml solvent) from % w/w

$$C_{vsolvent} = \frac{C_w}{100 - C_w} \times 100. \qquad \text{SI Equation 2.}$$

To illustrate the difference in values given by these methods for reporting concentration, we present values for sucrose concentration for % w/v (g sucrose / 100 ml solution), % w/w and % w/v (g sucrose / 100 ml solvent) (SI Table 1).

SI Table 1. A comparison between the concentrations of sucrose solutions given as % w/v (g solute / 100 ml solution), % w/w (g solute / 100 g solution) and % w/v (g solute / 100 ml solvent).

Concentration (% w/v) (g sucrose / 100 ml solution)	Concentration (% w/w) (g sucrose / 100 g solution)	Concentration (% w/v) (g sucrose / 100 ml solvent)
0	0	0
10	9.6	10.7
20	18.6	22.9
30	27.0	36.9
40	34.8	53.3
50	42.1	72.8
60	49.0	96.2
70	55.5	124.9
80	61.7	161.2
90	67.6	208.4

References

Balfour NJ, Gandy S, Ratnieks FLW (2015) Exploitative competition alters bee foraging and flower choice. Behavioral Ecology and Sociobiology 69:1731–1738. https://doi.org/10.1007/s00265-015-1985-y

Bolten AB, Feinsinger P, Baker HG, Baker I (1979) On the Calculation of Sugar Concentration in Flower Nectar. Oecologia 41:301–304.

Brown M, Downs CT, Johnson SD (2008) Sugar preferences of nectar feeding birds – a comparison of experimental techniques. Journal of Avian Biology 39:479–483. https://doi.org/10.1111/j.0908-8857.2008.04394.x

Lide DR (2004) CRC Handbook of Chemistry and Physics, 84th edn. CRC Press.

Schmid S, Schmid VS, Zillikens A, Harter-Marques B, Steiner J (2011) Bimodal pollination system of the bromeliad *Aechmea nudicaulis* involving hummingbirds and bees. Plant Biology Suppl. 1:41–50.
